# Triggering the Resolution of Immune Mediated Inflammatory Diseases: Can Targeting Leukocyte Migration Be the Answer?

**DOI:** 10.3389/fphar.2019.00184

**Published:** 2019-03-01

**Authors:** Sophie J. Hopkin, Jonathan W. Lewis, Franziska Krautter, Myriam Chimen, Helen M. McGettrick

**Affiliations:** ^1^Institute of Cardiovascular Sciences, University of Birmingham, Birmingham, United Kingdom; ^2^Rheumatology Research Group, Arthritis Research UK Centre of Excellence in the Pathogenesis of Rheumatoid Arthritis, Institute of Inflammation and Ageing, University of Birmingham, Birmingham, United Kingdom

**Keywords:** leukocytes, migration, inflammation, resolution, PEPITEM, sphingosine-1-phosphate, glucocorticoids, 11-beta hydroxysteroid dehydrogenase

## Abstract

Leukocyte recruitment is a pivotal process in the regulation and resolution of an inflammatory episode. It is vital for the protective responses to microbial infection and tissue damage, but is the unwanted reaction contributing to pathology in many immune mediated inflammatory diseases (IMIDs). Indeed, it is now recognized that patients with IMIDs have defects in at least one, if not multiple, check-points regulating the entry and exit of leukocytes from the inflamed site. In this review, we will explore our understanding of the imbalance in recruitment that permits the accumulation and persistence of leukocytes in IMIDs. We will highlight old and novel pharmacological tools targeting these processes in an attempt to trigger resolution of the inflammatory response. In this context, we will focus on cytokines, chemokines, known pro-resolving lipid mediators and potential novel lipids (e.g., sphingosine-1-phosphate), along with the actions of glucocorticoids mediated by 11-beta hydroxysteroid dehydrogenase 1 and 2.

## Introduction

**FIGURE 1:**
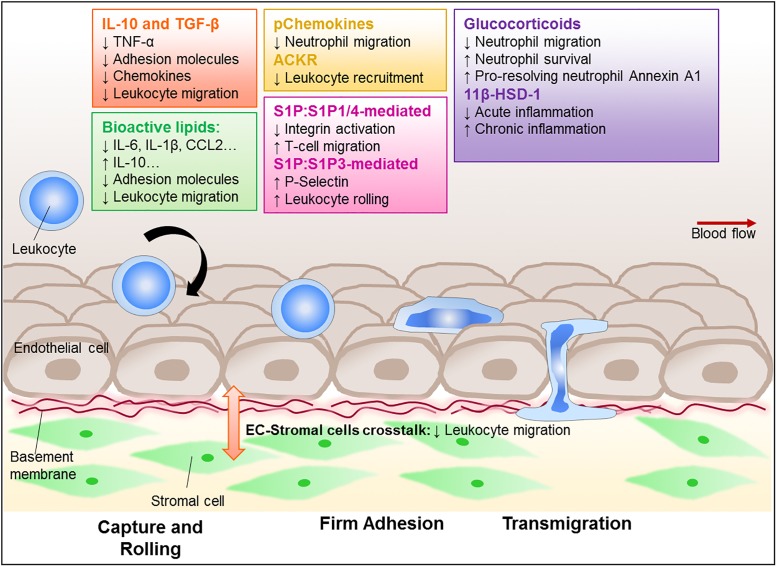
Regulation of leukocyte trafficking as a way to enhance resolution. Leukocytes, such as neutrophils, monocytes, and lymphocytes, are recruited to and migrate through the vessel wall to reach the site of inflammation. This process is tightly regulated and involves adhesion molecules and chemokine-chemokine receptor signal transduction, as well as interaction with the stromal compartment. Critically, these processes become dysregulated in chronic inflammatory diseases leading to aberrant recruitment that contributes to the diseases. There is a growing interest in finding ways to control leukocyte trafficking as a means to reduce the numbers of leukocyte in inflammatory sites, and therefore potentially allow resolution. Amongst those potential targets are cytokines, such as IL-10 and TGF-β; lipid mediators (e.g., lipoxins, resolvins, maresin-1, and S1P); annexin A1, glucocorticoids and its regulating enzyme 11β-HSD-1, as well as the recently characterized pChemokines and ACKRs. These potential targets are all capable of modulating leukocyte migration and further investigation is required to establish their potential pro-resolution roles.

Acute inflammation is a self-limiting, resolving response in which leukocyte entry and exit is tightly controlled. An imbalance in these processes permits accumulation and persistence of leukocytes within inflamed tissue, leading to damaging chronic non-resolving inflammation that underpins immune-mediated inflammatory diseases (IMIDs). Although significant advances have been made, we still do not fully understand the physiological processes regulating resolution of inflammation, and whether tissue-specific, or stimuli-specific processes exist. Current therapeutic strategies target leukocytes directly or their cytokine products, and hence the activation process of inflammation, rather than promoting resolution. Identifying the immune components that actively induce resolution of inflammation may be the key to novel therapeutic strategies for the treatment of IMIDs (Fullerton and Gilroy, 2016). In this review, we will focus on the pharmacological tools that influence the migration of leukocytes during an inflammatory response and whether such agents can trigger resolution (Figure 1).

## Leukocyte Trafficking in Health

Upon inflammation, blood vascular endothelial cells (EC) up-regulate adhesion molecules and chemokines necessary to support dynamic EC-leukocyte interactions and allow leukocytes to cross the EC barrier (Nourshargh and Alon, 2014; Mellado et al., 2015). The leukocytes themselves receive a series of sequential signals as they negotiate this barrier, which influence their adhesive and migratory properties, effector functions (Luo et al., 2015) and survival (Filer et al., 2006; McGettrick et al., 2006) at the inflamed site. At the blood-tissue interface, selectivity between neutrophils and T-cells arises from the production and use of specific capture receptors (E-, P-selectins, low affinity α_4_β_1_-integrin), adhesion molecules (β_2_-, β_1_-integrins binding ICAM-1 or VCAM-1, respectively), chemokine and junctional molecule combinations (Reglero-Real et al., 2016). Tissue-specific “address-codes” (Parsonage et al., 2005) are created by the interactions of tissue-resident stromal cells with neighboring EC (McGettrick et al., 2012) and provide an extra level of complexity to the leukocyte adhesion cascade, controlling the number and type of leukocytes recruited during a given inflammatory event.

## Chronic Inflammation: Dysregulation of Trafficking

Growing evidence indicates that leukocyte entry into, migration through and exit from peripherally inflamed tissues is changed to some degree in patients with IMIDs, and that these processes can differ between individual’s with the same clinical diagnosis, and over the life-time of disease [i.e., amongst different phases of disease, following therapeutic intervention; (Buckley and McGettrick, 2018)]. Susceptibility genes associated with IMIDs have been shown to directly influence leukocyte recruitment and migration. For instance, expression of the rheumatoid arthritis (RA) susceptibility variant of PTPTN22 (R620W) has been reported to increase the adhesive and migratory properties of murine T-cells (Burn et al., 2016) and human neutrophils (Bayley et al., 2015) in non-diseased models and subjects. Similarly alterations in the cellular metabolism of leukocytes [namely T-cells in RA (Shen et al., 2017) or monocytes in atherosclerosis] can render these cells hypermotile or overtly pro-inflammatory (Chimen et al., 2017). Additionally vascular EC from chronically inflamed tissues acquire pathogenic traits, including elevated expression of adhesion molecules (Jones et al., 1995; Salmi et al., 1997; Cañete et al., 2007). For instance, cultured rheumatoid synovial EC required minimum TNFα stimulation to recruit leukocytes (Abbot et al., 1999). Similarly, secretory smooth muscle cells associated with atherosclerotic plaques are able to prime neighboring blood vascular EC to recruit leukocytes in response to very low TNFα concentrations (Rainger and Nash, 2001). Pathogenic rheumatoid synovial fibroblasts overtly activate EC, leading to the inappropriate influx of leukocytes (Lally et al., 2005; Smith et al., 2008; McGettrick et al., 2009). These interactions evolve with the progression of RA (Filer et al., 2017). This will ultimately change the phenotype of EC (McGettrick et al., 2015) and therefore the types of leukocytes they recruited as the disease persists. Thus it is clear that IMIDs adversely affect key cellular components that control leukocyte migration. Whilst we currently are unable to modify the genetic background of a patient with IMIDs, targeting environmental alterations in key cellular components to trigger resolution pathways is a much needed strategy.

## Triggering Resolution of Immune-Mediated Inflammatory Diseases

In humans, it is difficult to evaluate the impact of current IMID therapies on the process of leukocyte trafficking, with many studies only commenting on the changes in cell numbers in one tissue. A reduction in leukocyte numbers at an inflamed site due to drug treatment could arise from (i) reduced entry, (ii) enhanced clearance, (iii) promotion of exit, or (iv) retention in lymph nodes or other peripheral tissues that result in reduced numbers of leukocytes in the circulation (e.g., S1P inhibitors *– see below*). Indeed, anti-cytokine therapies systemically target key molecules utilized during leukocyte trafficking, including EC activation (TNFα, IL-1β) or EC-stroma crosstalk (e.g., IL-6). Raising the question – what properties should a pro-resolving agent have? If we specifically focus on the context of leukocyte trafficking, potential modes of action would include, limiting cellular infiltration; inducing apoptosis; modulating chemokine and cytokine gradients to promote egress and clearance; reprogramming of leukocytes (e.g., macrophage) phenotypes to induce suppressor cells and induction of tissue repair mechanisms (Sugimoto et al., 2016a).

### Cytokines

The cytokine pathways promoting resolution are largely undefined thus far. Certain cytokines are considered to be anti-inflammatory, such as IL-10 and TGF-β, but does this mean they also induce resolution? IL-10 signaling caused the destabilization of TNFα and IL-1α mRNA, thereby reducing protein production in macrophages (Schaljo et al., 2009). Similarly, TGF-β can inhibit the translation of TNFα mRNA into protein in LPS-stimulated murine macrophages *in vitro* (Bogdan et al., 1992). Reduced TNFα levels at inflamed sites, as seen in patients treated with TNFα inhibitors, causes EC and stromal cells to revert to a resting-like phenotype, downregulating the expression of adhesion molecules and chemokines necessary to support leukocyte migration (Tak et al., 1996). Mice deficient in either IL-10 (Keubler et al., 2015) or TGF-β1 (Kulkarni and Karlsson, 1993; Dang et al., 1995; Letterio et al., 1996) have increased suspectibility to developing IMIDs. However, neither cytokine appeared to induce resolution when administered therapeutically in rodent models of IBD (Herfarth et al., 1998; Barbara et al., 2000; Kitani et al., 2000); and only TGF-β was shown to reduce leukocyte infiltration and disease severity (Kitani et al., 2000). Raising the question as to whether these cytokines can influence the migration of leukocytes to support resolution. IL-10 therapy has been reported to reduce the incidence of psoriasis relapse in a cohort of patients in remission (Friedrich et al., 2002), and induce clinical remission in ∼25% of patients with steroid-resistant Crohn’s disease when compared to placebo control (van Deventer et al., 1997). Whilst both cytokines modulate inflammation, their clinical potential as pro-resolving therapies has yet to be fully determined.

### Chemokines

Chemokines are active regulators of leukocyte migration into and out of tissues, as well as playing a key role in the positioning of leukocytes within the inflamed site. The successes and failures of targeting the chemokine pathway to block their pro-inflammatory functions and interfere with leukocyte migration has been reviewed elsewhere (Asquith et al., 2015). Recently, an alternative means of targetting chemokines to induce resolution was described: pChemokines are short-chain peptides with high affinity for chemokine glycosaminoglycan binding domain, which enables them to act as competitive inhibitors for chemokine receptors (McNaughton et al., 2018). pCXCL8 treatment was able to reduce neutrophil migration across CXCL8-treated endothelium *in vitro* and limited the numbers of leukocytes infiltrating arthritic murine joints (McNaughton et al., 2018). pChemokines are potentially a promising new therapeutic option for IMIDs, limiting the inflammatory infiltrate. It remains to be seen whether pChemokines also display other pro-resolving mechanisms, such as inducing tissue repair or reprogramming of macrophages from classical to alternative activation.

Endogenous removal of chemokines, either by drainage through the lymphatics or by chemokine-scavenging atypical chemokines (ACKRs), is necessary to facilitate the removal of the inflammatory infiltrate during resolution (Bonecchi and Graham, 2016). The potential role of ACKRs in resolution has been reviewed elsewhere (Bonecchi and Graham, 2016). As an example, ACRK2 (also known as D6) deficient mice have increased chemokine expression in the kidney (Bideak et al., 2018) and skin (Jamieson et al., 2005), accompanied by accumulation of T-cells in these tissues and exacerbation of nephrotoxic nephritis and psoriasis. To date, it is unclear whether the functional properties of ACKR2 can be defined as pro-resolving rather than anti-inflammatory, and whether ACKR2 has utility as a therapeutic target. Nevertheless, it is possible that agents that manipulate the expression and/or sequestering properties of ACKRs may be able trigger the resolution process in patients with IMIDs. Further work in this area is urgently required.

### Bioactive Pro-resolving Mediators – Resolvins, Lipoxins, Protectins, Maresin and Annexin A1

A variety of bioactive lipid mediators and proteins with pro-resolving properties have been identified, including lipoxins, resolvins, protectins and maresins (Serhan and Petasis, 2011), and subsequently shown to become dysregulated in patients with IMIDs contributing to pathology (Serhan, 2014; Brouwers et al., 2015). Circulating cytokine and chemokine levels can be directly modulated by such agents – for instance maresin can reduce IL-6, IL-1β (Marcon et al., 2013), and CCL2 levels (Martínez-Fernández et al., 2017), whilst annexin A1 (also known as lipocortin) is able to increase IL-10 production (Martínez-Fernández et al., 2017). Moreover, annexin A1, resolvins D1 and D2, and lipoxin A4 can all inhibit the expression of selectin molecules [e.g., P-selectin (Scalia et al., 1997), E-selectin (Chatterjee et al., 2014), or trigger L-selectin shedding (Strausbaugh and Rosen, 2001)] and also reduce β-integrin affinity states and their ability to cluster (Spite et al., 2009; Krishnamoorthy et al., 2010; Drechsler et al., 2015) on both leukocytes and on the endothelium. Reduced expression, activation and clustering of adhesion molecules, along with increased shedding will have considerable impact on the leukocyte recruitment cascade. Indeed, substantial evidence exists that pro-resolving lipid mediators, such as annexin A1, maresin-1, lipoxin A4, resolvin E1 and protectin D1 can inhibit neutrophil or monocyte infiltration into a variety of inflamed tissues, including mesentery (Lim et al., 1998), gut (Schwab et al., 2007), lung (Guido et al., 2013; Gong et al., 2014), brain (Gavins et al., 2012), atherosclerotic lesions (Drechsler et al., 2015), to promote resolution. Protectin D1, and to a lesser extent resolvin E1, are also able to enhance neutrophil and macrophage egress from inflamed cavities to neighboring lymphoid tissues (lymph node/spleen), further facilitating resolution through the removal of the microbial challenge via the lymphatics (Schwab et al., 2007). For further details, this topic is reviewed in depth elsewhere (Ortega-Gómez et al., 2013; Headland and Norling, 2015; Sugimoto et al., 2016b). Such data would indicate that these agents offer the potential to induce resolution in patients with IMIDs; however, the clinical efficacy of these agents has yet to be proven.

### Sphingosine-1-Phosphate

Numerous pharmaceutical companies are currently interested in modifying the bioactivity of sphingosine-1-phosphate (S1P) (Dyckman, 2017), yet it remains unclear whether S1P functions as a pro-resolving or a pro-inflammatory lipid mediator. The most abundant store of S1P is found in the blood, where the majority is bound to plasma proteins reducing its bioavailability (Christoffersen et al., 2011). The two main consequences of this are: (i) a S1P concentration gradient between the blood and tissue (Pappu et al., 2007) and (ii) reduced S1P receptor (SIPR) expression on circulating leukocytes (Lo et al., 2005). However, re-expression of surface S1PR1 and S1PR4 is stimulated by chemokine-induced integrin activation of T-cells bound to inflamed blood vascular EC, sensitizing these cells to S1P (Chimen et al., 2015). Under these circumstances, locally released S1P was able to inhibit T-cell transendothelial migration, by reducing the affinity state of β_2_-integrins from high to low (Chimen et al., 2015). In this study, B-cells recruited to the inflamed EC and binding adiponectin secrete a novel 14 aa immunomodulatory peptide, called PEPITEM (PEPtide Inhibitor of Transendothelial Migration) (Chimen et al., 2015). PEPITEM binds to cadherin-15 on the endothelium triggering S1P production and release through the S1P transporter, SPSN2 (Chimen et al., 2015).

The inability to produce PEPITEM, and thus stimulate local S1P production, contributes to the inappropriate accumulation of T-cells in inflamed tissues in type-1-diabetes and RA (Chimen et al., 2015). Thus in this context S1P acts in an anti-inflammatory manner and could be an early initiator of the pro-resolving machinery. Mast cell derived S1P can also indirectly regulate leukocyte rolling triggering rapid mobilization of P-selectin to the EC surface in a S1PR3 dependent manner in response to tissue damage (Nussbaum et al., 2015). In a counter-regulatory manner, leukocyte rolling was enhanced in S1PR1 deficient mice, indicating that S1PR1 is inhibitory for leukocyte rolling (Nussbaum et al., 2015). Thus the actions of S1P may be subtly modified dependent on the different S1PR triggered.

In addition to regulating leukocyte entry into inflamed peripheral tissues, S1P has also been reported to influence the transit through and exit from these sites across lymphatic endothelium (Ledgerwood et al., 2007). For instance, S1P reportedly enables activated CD4+ T-cells (OT-II cells) to persist and move about within inflamed ear pinnae when the cells are injected directly into the tissue, whereby inhibiting S1P signaling with fingolimod reduced the speed at which T-cells cells traveled within the ear (Jaigirdar et al., 2017). Moreover, fingolimod significantly reduced the number of activated T-cells retained in the ear, suggesting that in the absence of S1P signals the activated T-cells were now able to migrated out of the tissue across the lymphatics (Jaigirdar et al., 2017). Similarly, S1P signaling through S1PR1 blocked T-cell migration across lymphatic endothelial cells of the footpad (Ledgerwood et al., 2007), strongly indicating that S1P signaling regulates T-cell exit of peripheral tissues. Overall it appears that S1P has dual roles in regulating leukocyte recruitment and migration, acting to both promote and inhibit it depending on context. The relationship between these properties and the resolution processes remains to be fully elucidated.

Dysregulation of S1P production, leading to higher S1P levels in chronically inflamed tissues is a shared feature of many IMIDs. For example, elevated levels of the enzyme (SPHK-1) necessary for S1P synthesis have been reported in rheumatoid synovium (Jaigirdar et al., 2017) and ulcerative colitis (Karuppuchamy et al., 2017), whilst high concentrations of S1P occur in broncholavage fluid (Ammit et al., 2001) and cerebrospinal fluid (Kułakowska et al., 2010) from asthma and multiple sclerosis (MS) patients. This tends to support the notion that the bioactivity of S1P is pro-inflammatory rather than pro-resolving. Indeed, inhibiting S1P signaling with FTY720 has protective effects when administered therapeutically in rodent models of MS (Sheridan and Dev, 2014), inflammatory arthritis (Matsuura et al., 2000; Wang et al., 2007; Tsunemi et al., 2010; Fujii et al., 2012; Han et al., 2015), and systemic lupus erythematosus (SLE) (Wenderfer et al., 2008). Moreover, fingolimod is an FDA-approved treatment for MS with clinical efficacy in reducing relapses in patients with relapsing remitting MS (Kappos et al., 2006; Singer, 2013), but not those with primary progressive MS (Lublin et al., 2016). Nevertheless, existing S1P modulators are known to induce lymphopenia (Wang et al., 2007; Tsunemi et al., 2010) by blocking lymphocyte exit from lymph nodes (Mandala et al., 2002; Chiba, 2005; Han et al., 2015) – thus indirectly reducing the circulating numbers of cells available to enter peripherally inflamed sites. This can result in a general immunosuppression in patients; increasing susceptibility to opportunistic infections, whilst reducing vaccine efficiency (Massberg and von Andrian, 2006). Moreover, it is highly probable that S1P modulators also interfere with the S1P-dependent migration of T-cell out of peripherally inflamed tissues across lymphatic endothelium (Ledgerwood et al., 2007), and thus maybe responsible for retention of cells at the inflamed site further exacerbating disease.

### 11-Beta HSD Enzymes in Regulating GC Function

Glucocorticoids (GCs) are steroid hormones responsible for regulating cellular metabolism, immune function, adhesion molecule expression, and leukocyte migration (Tomlinson and Stewart, 2001). Dexamethasone (a synthetic GC) reduced the expression of E-selectin on inflamed aortic EC, disrupting neutrophil migration (Brostjan et al., 1997). By contrast, dexamethasone enhanced CXCL12-induced chemotaxis of resting human T-cells *in vitro* (Ghosh et al., 2009). Blocking GC function with prophylactic administration of glucocorticoid receptor (GR) antagonists exacerbated neutrophil infiltration into the synovial of carrageenan-induced monoarthritis in rats (Leech et al., 1998). GCs also influence cell viability, promoting neutrophil survival (Cox, 1995; Ruiz et al., 2002), whilst stimulating eosinophil apoptosis (Druilhe et al., 2003). Importantly, GCs can indirectly promote the resolution of inflammation through the induction of annexin A1 on human neutrophils and monocytes (Goulding et al., 1990). Annexin A1 can disrupt neutrophil migration, causing adherent neutrophils to detach from inflamed mesenteric endothelium and re-enter the circulation (Lim et al., 1998) restoring tissue homeostasis. Synthetic GCs clearly elicit cell-type specific effects, eliciting more immunomodulatory rather than immunosuppressive effects and may even exacerbate inflammation. Yet they are commonly used to treat IMIDs [e.g., RA, MS, psoriasis (Coutinho and Chapman, 2011)], where prolonged use is associated with metabolic and endocrine dysregulation (Schäcke et al., 2002).

The predominately active GC in humans is cortisol, which upon binding to the cytosolic GR, modifies gene expression to promote an anti-inflammatory response (Schüle et al., 1990; De Bosscher et al., 1997). The local bioavailability of GC is regulated by metabolic enzymes, including the two isoforms of 11β-hydroxysteroid dehydrogenase [11β-HSD-1 and 11β-HSD-2; (Seckl and Walker, 2001; Tomlinson and Stewart, 2001)]. Residing in the lumen of the ER, 11β-HSD-1 primarily reduces cortisone (inactive GC) to cortisol (active GC) increasing local active GC concentrations, whilst 11β-HSD-2 catalyzes the reverse reaction – inactivating cortisol and reducing active GC levels (Albiston et al., 1994; Seckl and Walker, 2001; Tomlinson and Stewart, 2001). 11β-HSD-1 expression and activity are ubiquitious, albeit at varying amounts: high expression is found in GC-target tissues [e.g., liver and fat; (Seckl and Walker, 2001)] and much lower levels are seen in leukocytes (Thieringer et al., 2001; Chapman et al., 2009; Coutinho et al., 2016). In contrast, 11β-HSD-2 expression and activity are largely restricted to mineralocorticoid-target tissues, e.g., the kidneys, pancreas and large intestine (Albiston et al., 1994), and not found in leukocytes. Importantly, the expression and activity of 11β-HSD-1 is dynamically regulated during inflammation, where cytokines such as IL-1β (Sun and Myatt, 2003), IL-4 (Thieringer et al., 2001), and IL-13 (Thieringer et al., 2001) induce 11β-HSD-1 activity stimulating local increases in active GC which exert anti-inflammatory and pro-resolving effects. Interestingly, GC metabolism is skewed in patients with IMIDs, such as SLE (Ichikawa et al., 1997) and RA (Hardy et al., 2006), toward cortisol production and therefore should trigger GC-induced anti-inflammatory/pro-resolving pathways to dampen the inflammatory response. However, despite elevated plasma cortisol levels in patients with IMIDs, the anti-inflammatory/pro-resolving GC pathways are not obviously triggered. This discrepancy has been attributed to an insufficient levels of active GCs, as this deficiency can be overcome by administration of high-dose GC mimics to IMID patients (Straub and Cutolo, 2016). Thus the relationship between plasma cortisol and active GC is not strictly linear in chronic inflammation, opening avenues for further research into the dysregulation of GC metabolism.

11β-HSD-1 have also been reported to modulate leukocyte trafficking by influencing expression of chemokines and adhesion molecules (Wamil et al., 2011; Kipari et al., 2013; Mylonas et al., 2017). However, *in vivo* studies blocking 11β-HSD-1 function with chemical agents or in 11β-HSD-1-deficient (*Hsd11b1*^-/-^) mice have reported conflicting findings. In a model of acute thioglycollate-induced peritonitis in mice, augmented leukocyte recruitment was observed following prophylactic inhibition of local 11β-HSD-1 (Coutinho et al., 2016) and in *Hsd11b1*^-/-^ mice (Coutinho et al., 2012). Similar findings were reported in carrageenan-induced pleurisy (Coutinho et al., 2012) and coronary artery ligation induced myocardial infarction (McSweeney et al., 2010) in *Hsd11b1*^-/-^ mice, supporting the concept that 11β-HSD-1 functions to limit inflammation. In contrast, lower amounts of MCP-1 were released by adipocytes from *Hsd11b1*^-/-^ mice on a high fat diet, resulting in fewer CD8^+^ T-cell and macrophage infiltrating mesenteric adipose tissue (Wamil et al., 2011). Similarly low VCAM-1 expression by aortic endothelial cells was attributed to the significant reduction in T-cell and macrophage within atherosclerotic plaques of *Hsd11b1*^-/-^ mice on high fat diet (Kipari et al., 2013). These studies indicate that 11β-HSD-1 activity promotes leukocyte recruitment and hence inflammation. The field currently believes that the functional outcomes of 11β-HSD-1 activity, whether these be pro or anti-inflammatory, is governed by a mixture of cell-specific, tissue-specific and inflammatory context-specific factors. Therefore, it is impossible to say with any certainty that 11β-HSD-1 has pro-resolving properties and is a viable drug target without further studies in this area.

That said, phase 2 clinical trials examining the efficacy of 11β-HSD-1 selective inhibitors, such as INCB13739 in obesity-related inflammatory diseases are ongoing (Anagnostis et al., 2013), but as yet no candidate drug is in the pipeline for IMIDs. Nevertheless caution is required: 11β-HSD-1 down-regulators [e.g., glycyrrhizic acid and rosiglitazone (Mai et al., 2007; Wake et al., 2007)] are associated with increased risk of cardiovascular-associated morbidity (Nissen and Wolski, 2007), hypertension encephalopathy (Russo et al., 2000) and hypokalemic paralysis (Pant et al., 2010). Given the tissue-restricted expression patterns of 11β-HSD, there is growing excitement about the potential to specifically modulate local GC concentrations using tissue-specific targeted therapies. However, we do not fully understand the role of these enzymes in specific IMIDs. Critically, the effects of endogenous GC and synthetic mimics are context dependent based on cell-type and local environmental conditions creating a complex interplay between GC, 11β-HSD enzymes and local environment, which is not yet fully understood. Clarifying the role of 11β-HSD enzymes in different IMIDs will allow the anti-inflammatory and pro-resolution properties that they exert to be exploited to promote the resolution of inflammation.

## Conclusion and Current Perceptions

The regulated movement of leukocytes into, through and out of peripheral tissues is vital in order to mediate tissue homeostasis in response to an inflammatory insult. We are expanding our understanding into how these processes are altered in the pathogenesis of IMIDs, and crucially the timing of such changes and their impact on the resolution of inflammation. With every step forward, key agents with the capacity to induce resolution and that may be amenable to therapeutic intervention become clearer. This represents an exciting new prospect that these novel drugs would actively target endogenous regulatory processes to reduce leukocyte entry into tissues and promote their clearance and egress to restore tissue homeostasis.

## Author Contributions

HM wrote the first draft of the manuscript. SH, JL, FK, and MC wrote sections of the manuscript. All authors contributed to manuscript revision, read and approved the submitted version.

## Conflict of Interest Statement

The authors declare that the research was conducted in the absence of any commercial or financial relationships that could be construed as a potential conflict of interest.
